# Alterations of Functional Brain Connectivity After Long-Duration Spaceflight as Revealed by fMRI

**DOI:** 10.3389/fphys.2019.00761

**Published:** 2019-07-04

**Authors:** Ekaterina Pechenkova, Inna Nosikova, Alena Rumshiskaya, Liudmila Litvinova, Ilya Rukavishnikov, Elena Mershina, Valentin Sinitsyn, Angelique Van Ombergen, Ben Jeurissen, Steven Jillings, Steven Laureys, Jan Sijbers, Alexey Grishin, Ludmila Chernikova, Ivan Naumov, Ludmila Kornilova, Floris L. Wuyts, Elena Tomilovskaya, Inessa Kozlovskaya

**Affiliations:** ^1^Laboratory for Cognitive Research, Higher School of Economics, Moscow, Russia; ^2^Institute of Biomedical Problems, Russian Academy of Sciences, Moscow, Russia; ^3^Radiology Department, Federal Center of Treatment and Rehabilitation, Moscow, Russia; ^4^Medical Research and Educational Center, Lomonosov Moscow State University, Moscow, Russia; ^5^Lab for Equilibrium Investigations and Aerospace, Faculty of Science, University of Antwerp, Antwerp, Belgium; ^6^iMec/Vision Lab, Faculty of Science, University of Antwerp, Antwerp, Belgium; ^7^Coma Science Group, GIGA Consciousness Research Centre, Neurology Department, University Hospital of Liège, Liège, Belgium; ^8^Gagarin Cosmonauts Training Center, Star City, Russia

**Keywords:** spaceflight, microgravity, cosmonauts, fMRI, functional connectivity, brain plasticity, vestibular function, support stimulation

## Abstract

The present study reports alterations of task-based functional brain connectivity in a group of 11 cosmonauts after a long-duration spaceflight, compared to a healthy control group not involved in the space program. To elicit the postural and locomotor sensorimotor mechanisms that are usually most significantly impaired when space travelers return to Earth, a plantar stimulation paradigm was used in a block design fMRI study. The motor control system activated by the plantar stimulation involved the pre-central and post-central gyri, SMA, SII/operculum, and, to a lesser degree, the insular cortex and cerebellum. While no post-flight alterations were observed in terms of activation, the network-based statistics approach revealed task-specific functional connectivity modifications within a broader set of regions involving the activation sites along with other parts of the sensorimotor neural network and the visual, proprioceptive, and vestibular systems. The most notable findings included a post-flight increase in the stimulation-specific connectivity of the right posterior supramarginal gyrus with the rest of the brain; a strengthening of connections between the left and right insulae; decreased connectivity of the vestibular nuclei, right inferior parietal cortex (BA40) and cerebellum with areas associated with motor, visual, vestibular, and proprioception functions; and decreased coupling of the cerebellum with the visual cortex and the right inferior parietal cortex. The severity of space motion sickness symptoms was found to correlate with a post- to pre-flight difference in connectivity between the right supramarginal gyrus and the left anterior insula. Due to the complex nature and rapid dynamics of adaptation to gravity alterations, the post-flight findings might be attributed to both the long-term microgravity exposure and to the readaptation to Earth’s gravity that took place between the landing and post-flight MRI session. Nevertheless, the results have implications for the multisensory reweighting and gravitational motor system theories, generating hypotheses to be tested in future research.

## Introduction

Recent advances in space vehicle engineering are expected to facilitate interplanetary space missions and space tourism. This will recruit what is known about the effects of space on the human body and mind, information which has been accumulating for more than a half century. Besides ionizing radiation, the most serious challenges for a human traveling to space are induced by microgravity. Body weightlessness and support unloading result in hypokinesia, vestibular sensory deprivation and an altered central interpretation of vestibular input (Young et al., 1984), as well as fluid redistribution (Smith et al., 1997), which in the aggregate cause detrimental effects on bones, muscles, cardiovascular function, neurovestibular function, and vision (Van Ombergen et al., 2017a). Such deterioration may progress into serious health issues and make long-term space flights impossible without proper microgravity countermeasures (Kozlovskaya et al., 2015; Shelhamer, 2015; Wang et al., 2018).

Besides issues that can be identified as health problems, microgravity induces many changes in behavior and performance, some of which may be considered as functional alterations and some as compensatory adaptations to the new environment (Newberg and Alavi, 1998; Bloomberg et al., 2015; Kornilova et al., 2017). The most pronounced among these changes are alterations in sensorimotor function, which, in its different aspects, recovers from days to weeks upon returning to the Earth (Lackner and DiZio, 1996). After a long-term space mission, many space travelers have difficulties standing upright and moving around just after landing (Clément et al., 2007). Later in post-flight period, the observed residual effects include disturbances in walking trajectories and postural stability, altered head position, tendency to raise arms to the sides, stamped gait or irregularly spaced steps (Mulavara et al., 2010), as well as increased reliance on visual feedback (Reschke et al., 1998) and increased time for motor task performance (Kubis et al., 1977; Kozlovskaya et al., 1982).

Alterations elicited by microgravity exposure are not limited to muscle “disuse” because of the weight unloading, but presumably affect all levels of the motor system and, according to the gravitational motor system theory, may be considered a dysfunction of the gravitational mechanisms in the motor system which provide reliability, accuracy, and stability of motor responses on the Earth’s surface (Kozlovskaya et al., 1988). These mechanisms are studied in human participants and animal models in actual spaceflight settings and in ground-based analogs, such as parabolic flight (PF), dry immersion (DI), and head-down bed rest (HDBR) (Watenpaugh, 2016; Van Ombergen et al., 2017a; Tomilovskaya et al., 2019). Up to now, an extensive body of empirical evidence suggests that a cascade of motor system modifications in microgravity is triggered by the lack of support afference from deep skin mechanoreceptors (Kozlovskaya et al., 2007). The reorganization of the motor system elicited by the lack of support is complemented by changes in biomechanics such as altered relationships between the mass of a body part and the force required to move it, and by the degraded performance of vestibular and proprioceptive sensory feedback (Reschke et al., 1998) which results in a conflict in the input from different sensory modalities (vestibular, proprioceptive, and visual) (Kornilova and Kozlovskaya, 2003; Davis et al., 2008). Altogether, such changes lead not simply to the prioritization of visual feedback for motor control, but to a necessity to rebuild the motor act coordination in order to find new adaptive solutions to the degree-of-freedom problem (Bernstein, 1967). Results consistent with this view and suggesting a gradual reinterpretation of muscle proprioceptive signals during prolonged exposure to microgravity (Lackner and DiZio, 1996) have been obtained, for example, in research on functional synergies during spaceflight (Clément et al., 1984; Massion, 1992). Similar motor learning processes were documented not only in the posture and locomotion domain, but also in manual tasks (Sangals et al., 1999; Bock et al., 2003; see Seidler et al., 2015 for a review).

In light of the previous discussion, the idea that alterations of sensorimotor functioning after microgravity exposure are very likely to reflect not only peripheral, but also central nervous system modification (or brain plasticity) seems logical and even commonplace. It has also received extensive support from animal models (Krasnov and Dyachkova, 1990; Holstein et al., 1999; DeFelipe et al., 2002; Dyachkova, 2007). But although a number of studies were conducted to collect evidence for microgravity-induced plasticity of human brain functioning with electroencephalography (EEG), the results were interpreted with caution because effects attributed to central neuroplasticity were hard to disentangle from numerous low-level confounds introduced by the complex nature of both the space mission environment and the observed physiological effects (Marušč et al., 2014).

Structural and functional magnetic resonance imaging (MRI) is seen as a more perspective method to reveal the mechanisms of space-induced neuroplasticity, although the MRI data may also be contaminated by side effects such as fluid shifting to the upper body (Roberts et al., 2015). Early calls for neuroimaging studies to find signs of neuroplasticity evoked by the adaptation to the space station environment (Newberg and Alavi, 1998) had little effect for almost two decades.

The first evidence for structural changes in the human brain after the long-term spaceflight includes a narrowing of the central sulcus, a shrinking of the cortico-spinal fluid (CSF) spaces at the vertex, and an upward shift of the brain within the skull as revealed by a clinical assessment of MRI scans (Roberts et al., 2017). Quantitative approaches have also shown extensive but focal bilateral decreases in gray matter volume in the temporal and anterior frontal cortices, as well as in the occipital cortex (Koppelmans et al., 2016; Van Ombergen et al., 2018); a focal increase in gray matter volume in the medial paracentral lobule was also observed (Koppelmans et al., 2016). The results of the only three microgravity-analog (HDBR) studies performed to the date partially corroborated these findings (Li et al., 2015; Roberts et al., 2015; Koppelmans et al., 2017b). As for the white matter, volumetric studies have shown a reduction of white matter volume in the left temporal lobe (Van Ombergen et al., 2018), and diffusion MRI data have indicated structural connectivity disruption in the longitudinal fasciculus, the inferior fronto-occipital fasciculus and the corticospinal tract in the right hemisphere, as well as in both inferior cerebellar peduncles (Lee et al., 2019).

The only published functional MRI (fMRI) study of a crew member after an actual spaceflight (compared to the preflight baseline) is a case study of a cosmonaut who spent 169 days on the ISS (Demertzi et al., 2016). The study used both task-based and resting-state fMRI techniques. While conventional task-based fMRI requires a participant to receive stimulation or to perform a motor or cognitive task while laying in the scanner, resting-state fMRI requires only relaxed wakefulness without any explicit stimulation or task being administered. The resting-state fMRI data are only used for analysis of intrinsic brain connectivity, especially within large-scale neural networks; the task-based fMRI data may be used for both activation and connectivity analyses. In the cosmonaut, the task-based analysis revealed higher activation in the supplementary motor area during the performance of an imaginary tennis task post-flight compared to preflight. The resting-state fMRI demonstrated reduced post-flight intrinsic connectivity in the right insula and between the left cerebellum and the right motor cortex (Demertzi et al., 2016).

Findings from over 10 microgravity analog studies published so far are very diverse, mainly due to the wide spectrum of study techniques or objectives (Roberts et al., 2010; Liao et al., 2012, 2013, 2015; Rao et al., 2014; Zhou et al., 2014; Cassady et al., 2016; Yuan et al., 2016, 2018a, b; Van Ombergen et al., 2017d). However, many of the brain regions that demonstrate microgravity-induced functional changes are associated with motor, vestibular and proprioceptive functions, or cognitive control (see Van Ombergen et al., 2017c for an extensive review).

Overall, the existing evidence advances the brain sensorimotor system and its connectivity with visual, vestibular, and proprioceptive brain regions as the primary target of neuroimaging research in microgravity-induced neuroplasticity. The ongoing prospective longitudinal studies that use different MRI methods (Koppelmans et al., 2013; Van Ombergen, 2017; Yuan et al., 2017) will be an important source of reliable evidence on this topic. The present paper reports preliminary results of an ongoing longitudinal task-based fMRI study devoted to the effects of long-duration spaceflight on cerebral motor function in humans. The research was conducted within the framework of the ESA/Roscosmos Brain DTI/Tractographia Project. To the best of our knowledge, it is the first published prospective controlled group fMRI study of large scale neural network plasticity in space travelers, and the first task-based functional connectivity MRI study of the effects of microgravity.

In this experiment, we compared brain activation and connectivity elicited by plantar stimulation in two groups: cosmonauts before and after long-term spaceflight, and healthy controls scanned twice with a comparable interval. Plantar stimulation produces support afference, which is believed to be a crucial factor for upright posture and normal terrestrial locomotion in humans (Layne et al., 1998; Kozlovskaya et al., 2007). During a long-term spacelight, prolonged support unloading takes place, which we expect to manifest in alterations of the functional connectivity between the brain areas contributing to motor control. Therefore, we hoped to elucidate those specific neural substrates that correspond to the postural and locomotor disturbances observed in cosmonauts upon re-entry to Earth. We also looked for correlations between the connectivity data and individual differences in the severity of the space motion sickness, which is believed to arise from the sensory conflict and to reflect the sensorimotor adaptation processes in microgravity (Heer and Paloski, 2006; Kornilova et al., 2013).

## Materials and Methods

### Participants

Eleven Russian cosmonauts and healthy age- and gender-matched volunteers (11 men not involved in the space program) took part in the study. At the time of the first exam the mean age of the participants was 45 years old (*SD* = 5) for the cosmonauts and 44 (*SD* = 6) for the control group. The study was approved as a part of the Brain DTI project by Committee of Biomedicine Ethics of the Institute of Biomedical Problems of the Russian Academy of Sciences and the Human Research Multilateral Review Board (HRMRB) according to the 18th World Medical Assembly of Helsinki, Finland, June 1964, amended by the 41st Assembly, Hong Kong, September 1989. All participants gave written informed consent for the study at enrollment.

In cosmonauts, subjective space motion sickness symptoms during space flight were assessed by a questionnaire first introduced at the MIR orbital station during the ‘ANKETA’ experiment (Kornilova, 1997) and since then used at the Institute of Biomedical Problems at the Russian Academy of Sciences. The cosmonauts were interviewed after the spaceflight before their vestibular tests on the first or 2nd day post-landing. Space motion sickness symptoms comprised complaints about orientation illusions, dizziness, poor coordination, difficulties in gaze fixation and tracing visual objects, nausea and vomiting (see the full list of questions in Supplementary Materials). The combination, intensity, and duration of these reactions were qualified according to the classification accepted in Russia (Kornilova et al., 2002, 2013). For the purpose of the present study, points were ascribed to each cosmonaut’s state after the flight: 0 corresponds to no complaints; 1 corresponds to moderately pronounced and rather short SMS syndrome; and 2 corresponds to complaints of pronounced and long lasting SMS syndrome with strong dizziness, vomiting, and discoordination. All control participants of the present study were ascribed zero points on this scale.

### Study Design

A 2 × 2 experimental design was used, with group (cosmonauts vs. controls) as a between-subject factor and session (post-flight vs. pre-flight) as a within-subject factor (repeated measures). In the control group, the first scanning session was treated as ‘preflight,’ and the second as ‘post-flight.’ According to the study design, only significant Group × Session effects were attributed to spaceflight.

Each cosmonaut was scanned before and after completing a long-term mission to the International Space Station between 2014 and 2017. For one cosmonaut, the data were aggregated across two separate space missions (and counted as the data from one subject). The control participants were scanned twice with comparable time intervals between the scans. A detailed description of demographics and timing data is presented in Table 1.

**TABLE 1:** General demographics, spaceflight-related information, and scan-to-scan intervals for the cosmonauts and control participants.

**Parameter**	**Cosmonauts**		**Controls**		**Difference**
	***Mean***	***SD***	***Mean***	***SD***	***p-value***
Age at the first scan (years)	45	5	44	6	0.680
Days to launch at the first scan	94	36			
Days after landing at the first scan	9.4	2.4			
Scan-to-scan interval (days)	282	63	249	57	0.212
Prior spaceflight experience (missions)	1.1	1.2			
Mission duration	183	55			
Space motion sickness score (‘ANKETA’)	0.72	0.64	0	0	

### The KORVIT System

**FIGURE 1:**
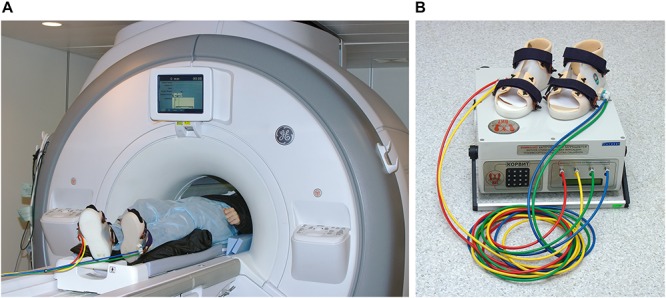
Experimental setup. **(A)** A participant positioned in the scanner wearing the KORVIT system boots. **(B)** Components of the KORVIT system.

The pneumatic KORVIT system (VIT, Saint Petersburg, Russia and Center of Aviaspace Medicine, Moscow, Russia) was used for mechanical stimulation of the soles support zones. The device was initially developed in the Institute of Biomedical Problems at the Russian Academy of Sciences (Grigor’ev et al., 2004; Kozlovskaya et al., 2007; Layne and Forth, 2008) as a mean of compensation of support afferentation deficit and therefore as a countermeasure to microgravity-induced motor impairment. The main component of the system is a pair of plastic boots with inflatable chambers mounted into the boot soles under the metatarsal and the heel zone. This layout of the chambers ensures stimulation of the zones with the highest density of mechanoreceptors and therefore elicits maximal response in the tonic muscle system. The chambers are connected to an air compressor by plastic cables. All parts of the system except for the air compressor are MR safe; during the scanning session the boots and cables were located in the scanner room, while the air compressor was located and operated upon in the scanner console room. The setup is illustrated in Figure 1. In the present study, the gait-like simulation mode was used with alternating pressure of 40 kPa cyclically administered to four zones on participants’ feet (right heel, right toes, left heel, left toes) with the frequency equal to 75 steps per minute.

According to previous studies, this mode of support stimulation leads to an extensive activation of the sensorimotor cortex (SMC) that controls locomotion (Kremneva et al., 2013). It has also been shown that this paradigm does not reflect actual locomotion, because the person does not make any movements. However, it has been proven that the afferent impulses induced by rhythmic stimulation of the support zone of the sole at a frequency and load similar to those experienced during real walking play an important role in supraspinal control (Kozlovskaya et al., 2007). Moreover, these results are similar to the results of other research groups that used imaginary walking paradigms (Kremneva et al., 2013).

### MRI Data Acquisition

All participants were scanned with a 3T GE Discovery MR750 scanner equipped with a 16-channel head, neck and spine (HNS) array coil. The scanner was located at the Federal Center of Medicine and Rehabilitation in Moscow, Russia.

For each participant, 160 T2^*^-weighted functional images were acquired in a single session of the gait-like plantar stimulation paradigm. Four extra volumes were scanned and automatically discarded by the scanner software prior to the acquisition of the functional data in order to achieve magnetic equilibrium. The gradient-echo echo-planar imaging (GRE EPI) pulse sequence was used with the following parameters: TR/TE/FA = 2000 ms/30 ms/77°, FoV = 192 mm × 192 mm × 126 mm, matrix size = 64 × 64 × 42, isotropic voxel size 3 mm. Each volume covered the whole brain with slices oriented parallel to the AC/PC line. The session lasted for approximately 5 min and was administered within the final part of the over 1 h long assessment that included both structural and functional scans (BRAINDTI project protocol). T1-weighted structural images were acquired in the first part of this program using the 3D fast spoiled gradient-echo (FSPGR) pulse sequence with an isotropic voxel size of 1 mm (TR/TE/FA = 7.9 ms/3.06 ms/12°).

### Procedure

During the plantar stimulation, a participant would lay in the scanner supine (head first) with KORVIT boots on his feet. He was instructed to keep his head still during the plantar stimulation. Extra foam padding was used to prevent excessive head motion elicited by the pulses of air pressure on the feet. The gait-like stimulation was administered in a block design with alternating 20-s blocks of two conditions: ‘stimulation’ and ‘rest.’ The stimulation cycle started with the rest period and was repeated eight times.

### Task-Based Activation Data Analysis

Data processing was performed with SPM 12 (Wellcome Institute of Cognitive Neurology^1^) and GLMFlex2 software (Aaron Shultz^2^). Preprocessing included the following steps: slice timing correction; realignment; longitudinal spatial coregistration of structural images from the two scanning sessions; spatial coregistration of the mean structural image and functional images; segmentation of the average structural volume into six tissue volumes; normalization into Montreal Neurological Institute (MNI) space; and spatial smoothing of the functional images with a Gaussian kernel of 8 mm full width at half-maximum (FWHM). Six residual head motion parameters (three for translation and three for rotation) were extracted during the realignment step.

To reveal the task-based activation, data were modeled using the general linear model as implemented in SPM12 software. For each participant, the MR signal was modeled using the canonical hemodynamic response function (HRF) with temporal derivatives (Friston et al., 2007). Temporal derivatives of the HRF were included into the model to prevent possible artifactual differences between groups or conditions. This concern was raised because the influence of spaceflight on the neurovascular coupling and HRF parameters is not yet known, but some effect is highly plausible given the alterations in cardiovascular functioning in space (Zhu et al., 2015) and especially the alterations of brain hemodynamics (Kawai et al., 2003; Blaber et al., 2013; Taylor et al., 2013) due to fluid shifting to the upper body. The data were analyzed as a block design with one experimental (plantar stimulation) condition while the baseline condition was not explicitly modeled to avoid model redundancy. Six parameters describing the head motion throughout the experimental session were included into the model as nuisance regressors. *T*-test contrasts for the BOLD signal change evoked by the plantar stimulation were obtained from both HRF and HRF temporal derivative regressors and combined into a single image for further use in the second-level analysis with the ‘derivative boost’ method (Lindquist et al., 2009; Steffener et al., 2010) implemented in the *spmup_hrf_boost* script (Pernet, 2014). A 2 × 2 ANOVA with group (cosmonauts vs. healthy controls) as a between-subject factor and session (pre-flight vs. post-flight) as a within-subject factor was performed as a second-level analysis scheme with the GLMFlex2 software. The results were assessed with a FDR-corrected cluster-wise threshold of *q* = 0.05 based on an uncorrected voxel-wise threshold of *p* = 0.001. Lenient statistical thresholds (*p* < 0.05 uncorrected, *k* = 5) were additionally applied to the group activation map obtained from the data of all 22 participants (both experimental and control groups) in order to define ROIs and the sensorimotor system mask for subsequent connectivity analyses. Peak activation coordinates were labeled with either FSL Harvard-Oxford maximal likelihood cortical and subcortical atlases (Desikan et al., 2006) or the AAL atlas (Tzourio-Mazoyer et al., 2002) for the cerebellum.

### Task-Based Connectivity Data Preprocessing

CONN Functional Connectivity Toolbox v. 17a (Whitfield-Gabrieli and Nieto-Castanon, 2012^3^) was used for the task-based connectivity preprocessing and further statistical analysis. Several standard procedures in the Conn toolbox were applied to the data already preprocessed for the task-based activation analysis in order to account for the residual motion-induced artifacts and physiological noise (denoising). First, head motion artifact detection was performed with the Artifact Detection Toolbox (ART^4^). Medium level thresholds that result in rejecting 3% of the normative sample data were applied; images demonstrating scan-to-scan head motion of more than 0.9 mm or global mean intensity change of more than 5 SDs were considered outliers. Outliers were subsequently included as nuisance regressors into the denoising linear model along with the residual head motion parameters. Another set of nuissance regressors was introduced by the anatomical component-based noise correction technique (aCompCor; Behzadi et al., 2007). With this method, noise ROIs are defined within the white matter and CSF masks individually segmented for each participant. The signal from the noise ROIs is decomposed with a principal component analysis (PCA) and time courses of the resulting components are regressed out from the data. The main BOLD-signal effects of the plantar stimulation and rest blocks were also regressed out to restrict the analysis to within-condition connectivity alterations rather than global changes of the correlation evoked by the task onset or offset. The linear detrending term was also applied. Then a standard temporal high-pass filter with a cutoff of 0.008 Hz was applied on the time series in order to further restrict the analysis to signal fluctuations which characterize task-based fMRI BOLD frequency band.

Two approaches to the task-based functional connectivity analysis were taken: voxel-to-voxel (data-driven) and ROI-to-ROI (hypothesis-driven). Follow-up hypotheses-driven seed-to-voxel analyses were also used to aid interpretation of the results. The connectivity results were labeled with the Harvard-Oxford and AAL atlases as well as the activation data.

### Task-Based Connectivity Voxel-to-Voxel Analysis

Firstly, voxel-to-voxel analysis was conducted to obtain the intrinsic connectivity contrast (ICC) values for each voxel in the whole brain. ICC was computed as a mean absolute value of the correlations of the time series for a given voxel with all other voxels included in the analysis (Martuzzi et al., 2011). The ICC values for each participant and condition (pre-flight and post-flight plantar stimulation and rest) were further used in the second-level ANCOVA with Group (cosmonauts vs. healthy controls) as a between-subject factor and session (post-flight vs. pre-flight) as a within-subject factor. Because of the imperfect cerebellum coverage in several scans, a concern was raised that the difference in the cerebellum ROIs between the preflight and post-flight sessions in the affected participants might also result in between-session differences in connectivity values. To account for this potential confound, the mean-centered difference in the total volume of the cerebellar network ROIs (defined with the Conn network atlas) for each participant was included in the analysis as a between-subject covariate. A Group × Session interaction was assessed with a FDR-corrected cluster-wise threshold of *p* = 0.05, *q* = 0.05 based on an uncorrected voxel-wise two-sided threshold of *p* = 0.001.

### Task-Based Connectivity ROI-to-ROI Analysis

An exploratory ROI-to-ROI analysis was adopted to test for the possible effects of long-duration spaceflight. The set of ROI selected for the analysis covered the sensorimotor, visual, proprioceptive, and vestibular brain systems thus including all main sources of afference utilized by the motor control system.

First, we included eight clusters identified in the activation map obtained from the plantar stimulation versus rest contrasts in all participants (cosmonauts and controls) at the liberal statistical threshold, obtained as described above (Table 3). Then all clusters from the sensorimotor, cerebellar, and visual systems from the Conn Networks atlas were also included. Since the insula plays an important role in motor, vestibular and proprioceptive functions, we included the ROIs for the right and left anterior and posterior insula (outlines from Kelly et al., 2012 were used). The bilateral ROIs for the thalamus, which is believed to be a relay station for vestibular signals (Lopez and Blanke, 2011), and the putamen, which is believed to play a crucial role in proprioception (Goble et al., 2012), were taken from the Harvard-Oxford atlas. Other ROIs were constructed with the MarsBar toolbox (Brett et al., 2002) as spheres around coordinates taken from the literature. The selection of the vestibular ROI replicated that by Van Ombergen et al. (2017b), with a different outline of the thalamus ROI as the only exception. Besides the insula and thalamus, vestibular ROIs included the right parietal operculum area 2 (rOP2), which is currently believed to represent the human vestibular cortex; the precuneus; the inferior parietal lobule, which is believed to be a part of the multimodal vestibular cortex (Zu Eulenburg et al., 2012; Dieterich and Brandt, 2015); and the bilateral vestibular nuclei (Kirsch et al., 2016). Proprioception ROIs were selected on the basis of data presented by Goble et al. (2012) and, besides the putamen, included the inferior frontal gyrus (IFG) and the inferior parietal cortex (BA40) bilaterally. The initially selected set of ROIs was further cross-checked for intersections, and only one of the two ROIs were included if a significant overlap was found (for example, the spherical ROI for the rOP2 was almost entirely covered by the activation-based rOP ROI). Since only differential contrasts of connectivity across groups and conditions were further considered, the residual marginal ROI overlap was not taken into account. Characteristics of the 27 areas forming the resulting ROI set are presented in Table 2.

**TABLE 2:** Characteristics of the ROIs selected for the ROI-to-ROI analysis of the functional connectivity between the sensorimotor, visual, proprioceptive, and vestibular systems.

**ROI**	**System**	**Source/reference**	**Shape**	**Center of mass MNI coordinates**		
				***x***	***y***	***z***
SensoriMotor. Lateral (L)	Sensorimotor	Conn Networks	Cluster	−55	−12	29
SensoriMotor. Lateral (R)	Sensorimotor	Conn Networks	Cluster	56	−10	29
SensoriMotor. Superior	Sensorimotor	Conn Networks	Cluster	0	−31	67
Cerebellar.Anterior (Lobules VI–IX)	Cerebellar	Conn Networks	Cluster	0	−63	−30
Cerebellar.Posterior (Crus)	Cerebellar	Conn Networks	Cluster	0	−79	−32
Visual.Primary	Visual	Conn Networks	Cluster	2	−79	12
Visual.Ventral	Visual	Conn Networks	Cluster	0	−93	−4
Visual. Dorsal (L)	Visual	Conn Networks	Cluster	−37	−79	10
Visual. Dorsal (R)	Visual	Conn Networks	Cluster	38	−72	13
Anterior Insula (L)	Proprioception, vestibular	Kelly et al.,2012	Cluster	−35	12	−5
Posterior Insula (L)	Proprioception, vestibular	Kelly et al.,2012	Cluster	−38	−9	2
Anterior Insula (R)	Proprioception, vestibular	Kelly et al.,2012	Cluster	38	8	−5
Posterior Insula (R)	Proprioception, vestibular	Kelly et al.,2012	Cluster	39	−12	6
Putamen (LR)	Proprioception	Harvard-Oxford atlas, subcortical; Goble et al.,2012	Cluster	−25/26	0/2	0/0
Thalamus (LR)	Vestibular	Harvard-Oxford atlas, subcortical	Cluster	−10/11	−19/−18	6/7
IFG (L)	Proprioception	Goble et al.,2012	10-mm sphere	−49	13	5
IFG (R)	Proprioception	Goble et al.,2012	10-mm sphere	53	16	7
IPC.BA40 (L)	Proprioception, vestibular	Goble et al.,2012	10-mm sphere	−62	−48	40
IPC.BA40 (R)	Proprioception, vestibular	Goble et al.,2012	10-mm sphere	60	−44	48
Vestibular nuclei (LR)	Vestibular	Kirsch et al.,2016	Two 5-mm spheres (L and R)	−16/16	−36/36	−32/−32
Precuneus	Vestibular	Zu Eulenburg et al.,2012	10-mm sphere	0	−52	27
Operculum (L)	Sensorimotor, proprioception	Task-based activation	Cluster	−52	−31	22
Operculum (R)	Sensorimotor, proprioception, vestibular	Task-based activation	Cluster	53	−28	22
Parahippocampal Gyrus	Visual	Task-based activation	Cluster	17	−24	−15
Cerebellum-01	Cerebellar	Task-based activation	Cluster	2	−42	−8
Cerebellum-02	Cerebellar	Task-based activation	Cluster	−15	−37	−24
Cerebellum-03	Cerebellar	Task-based activation	Cluster	17	−37	−26

The signal from each ROI was extracted only from gray matter voxels of the unsmoothed functional volumes, in order to avoid any additional risk of contaminating the data with white matter or CSF signals or with signals from other ROIs. Then, the task modulation of the ROI-to-ROI functional connectivity was assessed for the ‘preflight’ and the ‘post-flight’ sessions with the individual general psychophysiological interaction model (gPPI; McLaren et al., 2012) for each participant. The resulting differential ROI-to-ROI connectivity values for the active plantar stimulation condition over the implicitly modeled baseline were further used in the second-level ANCOVA with group (cosmonauts vs. healthy controls) as a between-subject factor and session (pre-flight vs. post-flight) as a within-subject factor, and the mean-centered difference in the total volume of the cerebellar network ROIs as a covariate. The results were considered at the levels of (a) individual modified connection (two-sided *t*-test, analysis-level FDR-corrected, *p*_corr_< 0.05); (b) ROI demonstrating a modified connectivity pattern; and (c) network (clusters of modified connections). At the latter two levels we used network-based statistics, taking into account the connection intensity (NBS; Zalesky et al., 2010) for the correction for multiple comparisons (FDR at the ROI level and FWE at the network level, *p*_corr_< 0.05, cluster-defining threshold *p* < 0.05 uncorrected).

The NBS statistic utilizes the permutation test and may be understood as an analog of the topological correction for multiple comparisons in the connectivity domain (with individual connections in place of voxels and subnetworks as clusters of connections). Therefore the NBS statistics is more sensitive but less spatially specific than the more conventional analysis at the level of individual connection. Due to the nature of the NBS statistics, inference limitations apply: the subnetworks may only be discussed in their integrity; no individual connection change may be considered significant on the basis that it belongs to a significantly changing NBS network. As implemented in Conn, the ROI-level NBS analysis treats only connections originating from the ROI under consideration and involves an extra FDR correction for the number of ROIs.

### Follow-Up Seed-to-Voxel Analysis

To aid interpretation of the results, we performed a follow-up whole-brain seed-to-voxel analysis using as seeds the ROIs that demonstrated significant effects of Group × Scanning session interaction in the NBS ROI-to-ROI analysis (ROI level). While the NBS analysis does not allow for any inference regarding individual, pairwise connections between ROIs, the seed-to-voxel analysis aims at testing whether the observed connectivity alterations are diffuse in their nature or have any compact localizable addressees. We also performed a whole-brain seed-to-voxel analysis using as a seed the cluster revealed by the ICC analysis as sensitive to the cosmonaut vs. control, post- vs. pre-, task vs. baseline interaction.

In each seed-to-voxel analysis, we used the PPI connectivity data computed between the seed region and every brain voxel outside the seed region. These differential values were entered into the second-level ANCOVA, analogous to what was implemented for the ROI-to-ROI analysis: group (cosmonauts vs. healthy controls) as a between-subject factor, session (pre-flight vs. post-flight) as a within-subject factor, and the mean-centered difference in the total volume of the cerebellar network ROIs as a covariate. Seeds were tested one at a time.

Additionally, we conducted a set of second-level ANCOVAs, testing for possible correlations between the post-flight vs. pre-flight difference in connectivity of the seeds and the individual scores of space motion sickness severity (the mean-centered difference in the total volume of cerebellar network ROIs was also included as a covariate of no interest). Again, seeds were tested one at a time.

Besides the correction for multiple comparisons performed at the level of each resulting spatial map (cluster-wise topographic FDR correction; *p* < 0.05, *q* < 0.05; voxel-wise cluster-forming two-sided threshold *p* < 0.001 uncorrected), the statistical thresholds for the results were also corrected for the total number of seed-to-voxel analyses (*p* < 0.05/12 = 0.004).

## Results

### Assessment of Potential Confounds

There were no significant differences in the mean age of the groups at the first scanning session (see Table 1). No statistically significant differences were found between groups or sessions in the standard deviations of displacement or rotation across any of the six registered head motion parameters. The percentage of the scans classified by the ART toolbox as invalid varied from 0 to 8% per participant; the total of 1.22% scans were discarded as invalid for the entire dataset. No participant was excluded from the analysis due to excessive head motion. The scan-to-scan head motion before scrubbing averaged 0.15 mm, *SD*: 0.05 mm per participant with a mean maximum motion of 1.14 mm, *SD*: 1.28 mm. No effects of the group, session or interaction of these factors on the general voxel-to-voxel correlation (GCOR; Saad et al., 2013) were revealed.

### Brain Activation

**FIGURE 2:**
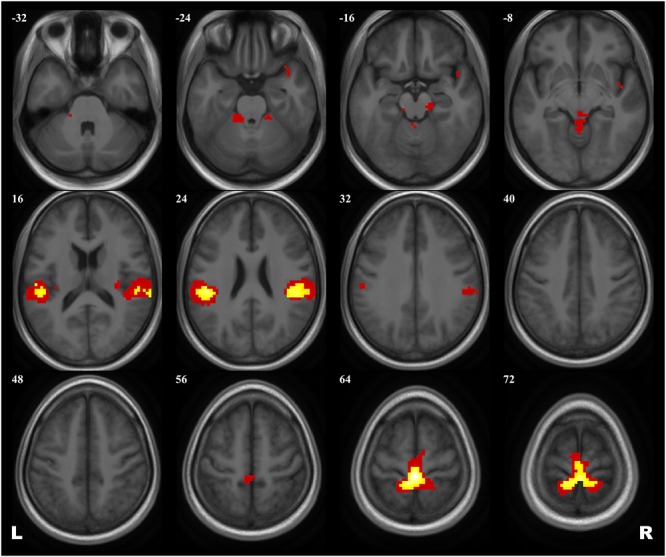
Group map of the activation elicited by plantar stimulation in all participants (both cosmonauts and healthy controls). Yellow areas depict activation at a reliable statistical threshold (cluster-wise FDR correction, *q* < 0.05, *p* < 0.05; voxel-wise cluster-defining threshold, *p* < 0.001 uncorrected). Activation at a lenient statistical threshold (*p* < 0.05 uncorrected voxel-wise, *k* = 5) is shown in red. The statistical images are presented as overlays upon the average structural image of all participants converted to the MNI space.

A 2 × 2 ANOVA showed no effect of Group × Scanning session interaction on brain activation evoked by the plantar stimulation paradigm. The aggregated statistical map for both groups revealed an activation pattern that included the primary SMC, the supplementary motor cortex (SMA), extensive regions in operculum bilaterally, and, when applying low statistical thresholds, areas of the right insula and the temporal pole and of the anterior cerebellum (see Figure 2 and Table 3).

**TABLE 3:** Clusters of activation revealed by the plantar stimulation in all participants.

**Cluster**		**Volume, voxels (mm^3^)**	***T*-statistics**	**MNI coordinates (center of mass/peak)**			**Region labels^+^**
				***x***	***y***	***z***	
**(1) SMC/SMA bilateral**	FDR_C_-corr.	181 (4887)		−1	−36	67	Precentral gyrus, post-central gyrus
	*p* < 0.05 uncorr.	430 (11610)		−1	−35	66	Post-central gyrus, precentral gyrus, precuneous cortex, superior parietal lobule
	
	Peaks		8.91^*^	0	−34	65	Post-central gyrus
			4.67^*^	−3	−19	68	Precentral gyrus
			4.23^*^	15	−40	71	Post-central gyrus
			3.31	6	−13	68	Juxtapositional lobule cortex (formerly supplementary motor cortex)

**(2) Right operculum**	FDR_C_-corr.	93 (2511)		51	−29	22	Parietal operculum cortex, planum temporale
	*p* < 0.05 uncorr.	308 (8316)		53	−28	22	Parietal operculum cortex, planum temporale, supramarginal gyrus (anterior division)
	
	Peaks		5.59^*^	45	−33	22	Parietal operculum cortex
			5.09^*^	66	−31	17	Superior temporal gyrus, posterior division
			4.92^*^	48	−22	20	Parietal operculum cortex
			3.31	32	−24	16	Insular cortex
			2.60	66	−19	17	Post-central gyrus

**(3) Left operculum**	FDR_C_-corr.	68 (1836)		−49	−30	21	Parietal operculum cortex, central opercular cortex
	*p* < 0.05 uncorr.	294 (7938)		−52	−31	22	Parietal operculum cortex, planum temporale, supramarginal gyrus (anterior division)
	
	Peaks		6.26^*^	−48	−31	23	Parietal operculum cortex
			3.83^*^	−54	−22	17	Central opercular cortex
			2.23	−33	−28	20	Central opercular cortex

**(4) Cerebellum-01**	*p* < 0.05 uncorr.	55 (1485)		2	−42	−8	Vermis III–IV, brain stem
	
	Peaks		3.68	0	−46	−10	Vermis IV–V
			3.11	0	−31	−4	Brain stem

**(5) Right temporal pole/insula**	*p* < 0.05 uncorr.	41 (1107)		42	6	−15	Temporal pole, planum polare, insular cortex
	
	Peaks		2.44	45	−4	−7	Planum polare
			2.21	39	11	−25	Temporal pole
			2.10	44	13	−12	Temporal pole

**(6) Cerebellum-02**	*p* < 0.05 uncorr.	36 (972)		−15	−37	−24	Left cerebellum III, IV–V lobules
	
	Peaks		2.83	−9	−40	−25	Left cerebellum III
			3.33	−18	−34	−25	Left cerebellum IV–V

**(7) Parahippocampal gyrus**	*p* < 0.05 uncorr.	11 (297)		17	−24	−15	Parahippocampal gyrus, posterior
	
	Peaks		2.55	15	−25	−16	Parahippocampal gyrus, posterior

**(8) Cerebellum-03**	*p* < 0.05 uncorr.	10 (270)		17	−37	−26	Right cerebellum IV–V lobules
	
	Peaks		2.50	21	−37	−28	Right cerebellum IV–V

^+^Only labels covering at least 5% of the cluster volume are listed. ^*^Peaks within the clusters that survived FDR_*C*_ correction.

### Voxel-to-Voxel Connectivity

**FIGURE 3:**
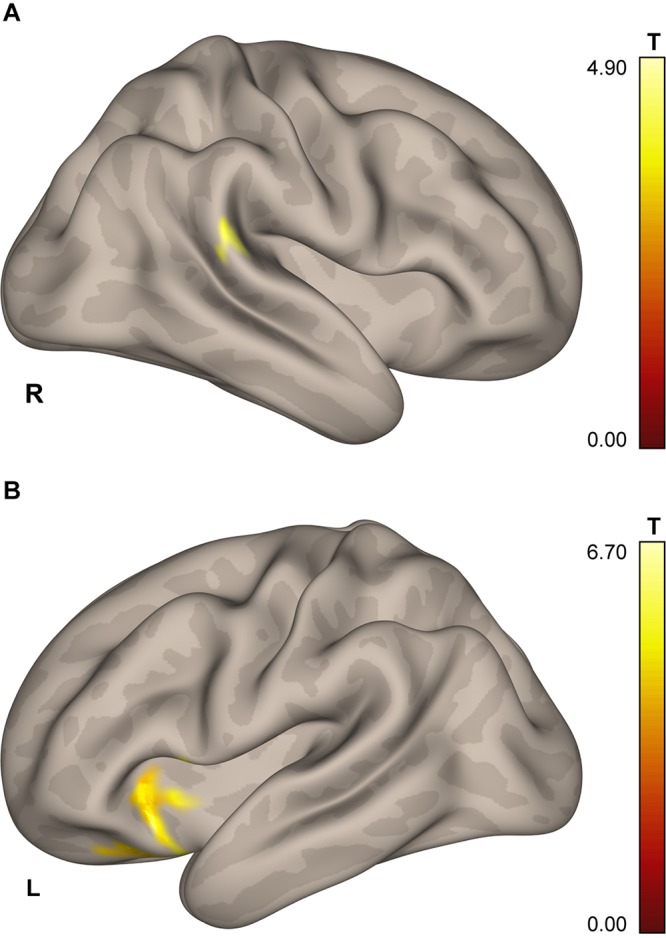
Results of the ICC and follow-up seed-to-voxel analysis. **(A)** Area in the lower portion of the right posterior supramarginal gyrus demonstrating higher connectivity with the rest of the brain for cosmonauts vs. controls, post- vs. pre-flight, plantar stimulation vs. rest, as revealed by the ICC analysis (cluster-wise FDR correction, *q* < 0.05, *p* < 0.05; voxel-wise cluster-defining threshold, *p* < 0.001 uncorrected). **(B)** Cluster in the left orbitofrontal cortex, insula and operculum, demonstrating correlation between the severity of space motion sickness and post- vs. pre-flight, plantar stimulation over the resting baseline difference in functional connectivity, with the seed in the rpSMG area shown in the **(A)**. Results are shown after the cluster-wise FDR correction, *q* < 0.05, *p* < 0.004 = 0.05/12; voxel-wise cluster-defining threshold, *p* < 0.001 uncorrected.

The whole-brain analysis revealed a cluster in the right posterior supramarginal gyrus (pSMG; 567 mm^3^; center of mass: *x* = 63, *y* = −37, *z* = 13; see Figure 3A) demonstrating the effect of a Group × Scanning session interaction for the ICC change during the task blocks over the baseline.

### ROI-to-ROI Connectivity

ROI-to-ROI analysis exploring the connectivity between the motor, somatosensory, visual, proprioceptive, cerebellar and vestibular brain systems, demonstrated significant post-flight alterations compared to the between-session differences observed in the control group.

Alterations were found at the levels of individual connection, ROI and subnetwork. The following individual connections showed modifications significant at the *p* < 0.05 threshold after analysis-level FDR-correction. Functional coupling between the right and left posterior insulae significantly increased in cosmonauts post-flight, while connections degraded between the posterior cerebellum and the primary visual cortex, and between the anterior cerebellum (activation cluster Cerebellum-03) and right parietal cortex (BA40).

**FIGURE 4:**
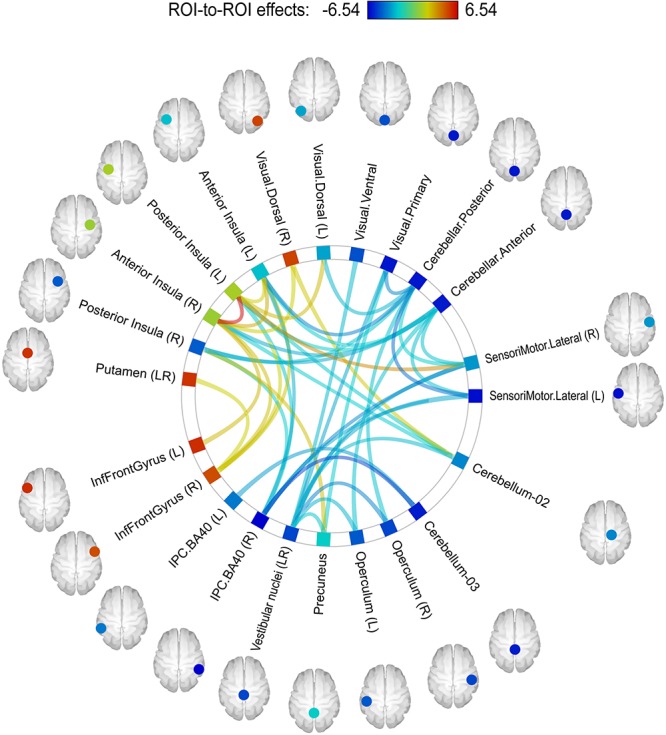
A subnetwork identified by the NBS network-level ROI-to-ROI connectivity analysis and demonstrating changes in PPI connectivity values post-flight vs. pre-flight, cosmonauts vs. controls. Line color indicates the sign and magnitude of the effect for individual connections comprising the network. The network-defining threshold (connection-wise) was set to *p* < 0.05, and the network-level results were FWE-corrected for multiple comparisons (NBS by intensity, *p* < 0.05 at the network level).

**FIGURE 5:**
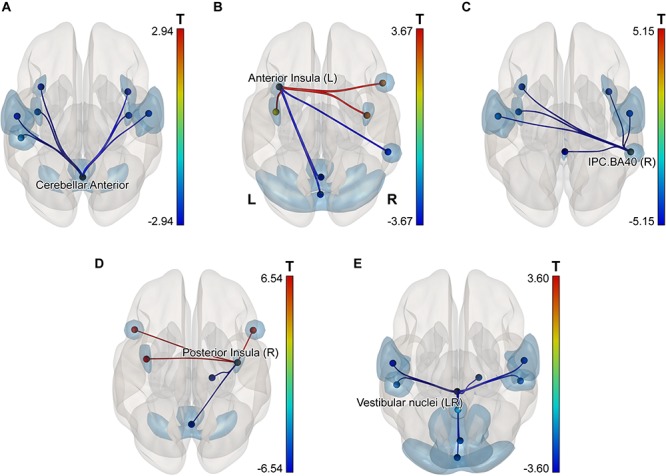
ROI-level networks showing significant PPI connectivity modifications in the NBS analysis post-flight vs. pre-flight, cosmonauts vs. controls for the following regions: **(A)** the anterior part of the cerebellar network (Conn Networks atlas); **(B)** the left anterior insula; **(C)** the right inferior parietal cortex, BA40; **(D)** the right posterior insula; **(E)** the vestibular nuclei. The network-defining threshold (connection-wise) was set to *p* < 0.05, and the ROI-level results were FDR-corrected for multiple comparisons (NBS by intensity, *q* < 0.05, *p* < 0.05 at the ROI level). Line color indicates the sign and magnitude of the effect for individual connections comprising the networks. Note that due to the nature of the NBS analysis, no individual connection contributing to the significantly changed network may be considered as significantly altered on its own. Cyan blobs indicate the location of the ROIs for the illustrative purposes.

In order to detect more subtle although less spatially specific changes, we computed ROI-level and network-level network-based statistics by intensity (NBS; Zalesky et al., 2010). The network-level NBS identified a subnetwork demonstrating effects of a Group × Scanning session interaction (at *p* < 0.05, FWE-corrected; see Figure 4). This subnetwork included five regions also showing significant connectivity modifications at the ROI level (at *p* < 0.05, FDR-corrected), namely: the vestibular nuclei (intensity = 22.88, size = 8), the right parietal cortex (intensity = 20.24, size = 6), the anterior part of the cerebellar network (Conn Networks atlas; intensity = 17.47, size = 7), the right posterior insula (intensity = 16.87, size = 5) and the left anterior insula (intensity = 16.47, size = 6). It is noteworthy that all suprathreshold connections of the vestibular nuclei, the right parietal cortex and the cerebellar network (anterior part) ROIs demonstrated a negative shift in PPI values (task vs. baseline differential connectivity) in cosmonauts post-flight vs. pre-flight. Connections comprising the altered networks of the affected ROIs are also presented in Figure 5 and Table 4.

**TABLE 4:** Results of the ROI-level NBS analysis (NBS by intensity).

**Analysis unit**	**Intensity**	***t*(19)**	***p*-unc.**	***p*-FDR**	***p*-FWE**
Network_1/1 (Size = 71)	204.24		0.0084		0.0086
Seed Vestibular Nuclei (VNLR)	22.88		0.0015	0.0397	0.0332
VNLR—Visual.Primary		–3.60	0.0019	0.0261	
VNLR— Operculum (R)		–3.58	0.002	0.0261	
VNLR—SensoriMotor.Lateral (L)		–3.04	0.0067	0.0578	
VNLR—Operculum (L)		–2.72	0.0137	0.0715	
VNLR—SensoriMotor.Lateral (R)		–2.71	0.0138	0.0715	
VNLR—Cerebellum-02		–2.63	0.0166	0.072	
VNLR—Visual.Ventral		–2.37	0.0284	0.1054	
VNLR—Precuneus		–2.22	0.0384	0.1249	
Seed Proprio.IPC.BA40 (R)	20.24		0.0031	0.042	0.0637
IPC.BA40 (R)—Cerebellum-03		–5.15	0.0001	0.0015	
IPC.BA40 (R)—SensoriMotor.Lateral (R)		–4.06	0.0007	0.0087	
IPC.BA40 (R)—Anterior Insula (L)		–3.49	0.0025	0.0214	
IPC.BA40 (R)—SensoriMotor.Lateral (L)		–2.84	0.0105	0.0686	
IPC.BA40 (R)—Anterior Insula (R)		–2.45	0.024	0.1246	
IPC.BA40 (R)—Posterior Insula (L)		–2.26	0.0355	0.1536	
Seed Cerebellar.Anterior	17.47		0.0065	0.0457	0.124
Cerebellar.Anterior—Anterior Insula (R)		–2.94	0.0084	0.1186	
Cerebellar.Anterior—Posterior Insula (R)		–2.90	0.0091	0.1186	
Cerebellar.Anterior—SensoriMotor.Lateral (L)		–2.52	0.021	0.1456	
Cerebellar.Anterior—Operculum (L)		–2.40	0.0269	0.1456	
Cerebellar.Anterior—SensoriMotor.Lateral (R)		–2.38	0.028	0.1456	
Cerebellar.Anterior—Anterior Insula (L)		–2.19	0.041	0.1682	
Cerebellar.Anterior—Posterior Insula (L)		–2.14	0.0453	0.1682	
Seed Posterior Insula (R)	16.87		0.0076	0.0457	0.1417
Posterior Insula (R)—Posterior Insula (L)		6.54	0.000	0.0001	
Posterior Insula (R)—Cerebellar.Anterior		–2.77	0.0123	0.107	
Posterior Insula (R)—IFG (L)		2.76	0.0123	0.107	
Posterior Insula (R)—IFG (R)		2.62	0.0167	0.1086	
Posterior Insula (R)—Cerebellum-02		–2.17	0.0426	0.1713	
Seed Anterior Insula (L)	16.47		0.0085	0.0457	0.1554
Anterior Insula (L)—Cerebellar.Posterior		–3.67	0.0016	0.0422	
Anterior Insula (L)—Posterior Insula (R)		2.94	0.0084	0.103	
Anterior Insula (L)—IPC.BA40 (R)		–2.78	0.0119	0.103	
Anterior Insula (L)—Cerebellar.Anterior		–2.49	0.0222	0.14	
Anterior Insula (L)—IFG (R)		2.40	0.0269	0.14	
Anterior Insula (L)—Posterior Insula (L)		2.19	0.0409	0.1772	

*The entire network revealed in the NBS analysis at the network level is comprised of 71 connections. The table is limited to connections of the seeds that demonstrated ROI-level effects significant at the *p* < 0.05 (FDR-corrected) level, NBS by intensity.*

### Seed-to-Voxel Connectivity

Regions identified in the NBS ROI-to-ROI and ICC results as sites involved into connectivity alterations due to the spaceflight were used as seeds for the follow-up seed-to-voxel analyses performed according to the same scheme as in the main analysis (cosmonauts vs. controls, post-flight vs. pre-flight). This approach aimed to check whether the observed connectivity changes are diffuse in their nature or have any compact localizable addressees across the whole brain.

The second set of the seed-to-voxel analysis employed the same six seeds as described above (five ROIs from the NBS results, and the rpSMG cluster from the ICC results). Now the post-flight vs. pre-flight differences in PPI values characterizing the connectivity of these regions with the rest of the brain were tested for significant correlations with the individual SMS scores.

The results showed a positive correlation of the severity of space motion sickness with differential post-to-preflight, task-to-baseline connectivity between the rpSMG seed and the left insular, opercular, and frontal orbital cortices (Figure 3B and Table 5; cluster-defining threshold *p* < 0.001, cluster-level FDR correction with correction for the total number of seed-to-voxel follow-up analyses: *p* < 0.05/12 = 0.004).

**TABLE 5:** Correlation between the severity of space motion sickness and seed-to-voxel connectivity.

**Seed**	**Resulting cluster center of mass, MNI**			**Cluster size, vx (mm^3^)**	**Cluster *p* uncorr.**	**Cluster *p* FDR-corr.**	FDR *p-*thresh^*^	**Region labels^+^**
	***x***	***y***	***z***					
rpSMG	−34	20	−9	165 (4455)	0.000	0.000	0.004	Frontal orbital cortex L Insular cortex L Frontal operculum cortex L

^+^Only labels covering at least 5% of the cluster volume are listed. ^*^Threshold, corrected for the total number of the seed-to-voxel follow-up analyses.

## Discussion

The present study reports on alterations of task-based functional brain connectivity in a group of 11 cosmonauts after spaceflight as compared to a healthy control group. To recruit the postural and locomotor sensorimotor mechanisms that are usually most significantly impaired when space travelers return to the Earth, a plantar stimulation paradigm was used in a block design fMRI study.

Task-specific functional connectivity modifications were revealed within a set of regions involving the sensorimotor, visual, proprioceptive, and vestibular neural networks. The most notable post-flight findings include an increase in stimulation-specific connectivity of the right posterior supramarginal gyrus with the rest of the brain (as revealed by the ICC measure); strengthened connections between the left and right insulae and decreased coupling of the cerebellum with the visual cortex and with the right inferior parietal cortex (BA40) (revealed by the connection-wise ROI-to-ROI approach); and altered connectivity of the bilateral insulae, vestibular nuclei, right inferior parietal cortex (BA40) and cerebellum with other areas associated with motor, visual, vestibular, and proprioception functions (revealed by the NBS approach). A correlation was also observed between the severity of space motion sickness symptoms and connectivity between the right posterior supramarginal gyrus and the left insular region.

Since no previous studies have reported data on task-based functional brain connectivity after an actual space flight, we are unable to perform a direct comparison of our results with the previous findings. However, our data are consistent with many aspects of the broader literature, including structural neuroimaging and microgravity analog research. At the same time, as shown by the example of the EEG, which is so far the only neuroimaging technique accessible both in space and in terrestrial settings, neurophysiological data from actual and simulated microgravity may be substantially inconsistent due to multiple factors such as details of the environment, stressors and emotional states that might contaminate the observed effects (Marušč et al., 2014; Van Ombergen et al., 2017c).

Given the lack of data available for direct comparison in the field, later in the discussion we introduce some speculations that we believe to be helpful for hypothesis generating and future research. We hope that subsequent progress in neuroimaging studies of microgravity would rule out some of the theories discussed below in favor of the others.

### Task-Based Activation

The task-based fMRI activation pattern evoked by the KORVIT plantar stimulation system in our study included the primary SMC, the SMA, the SII cortex (operculum) bilaterally, and, at a liberal statistical threshold, cerebellar and insular areas. Such a pattern is typical for passive gait-like plantar stimulation (Dobkin et al., 2004; Golaszewski et al., 2006; Kremneva et al., 2013; Jaeger et al., 2014; Labriffe et al., 2017) and is characterized by reduced insular and cerebellar activation compared to active foot stimulation paradigms (Sahyoun et al., 2004). KORVIT gait-like stimulation was applied to the sole zones with the maximal density of mechanoreceptors, and we believe that through proper modeling of the support afference our fMRI paradigm evoked the neural circuits essential for human upright posture and normal bipedal locomotion (Kozlovskaya et al., 2007) which might be modified in microgravity and, therefore, were the target of our research. At the same time, it should be noted that KORVIT gait-like stimulation does not activate the neural representation of the tonic muscle system exclusively. This is due to the involvement of other skin receptors, the low spatial resolution of the fMRI, and, last but not the least, the complex principles of individual muscle representation in the SMC (Kakei et al., 1999), resulting in a substantial overlap of different muscle projections (Melgari et al., 2008). Therefore, we refer to the pattern of activation obtained in our study as the neural correlates of plantar stimulation rather than the neural correlates of support afference, or tonic muscle system.

The present study failed to reveal any spaceflight-related significant differences in brain activation evoked by the plantar stimulation. In the presence of substantial individual variability among the cosmonauts (reported also by Roberts et al., 2010 in a pilot HDBR study), this null result may indicate insufficient statistical power. However, it may also mean that the ‘core’ neural system associated with the reaction to support loading remains intact after microgravity exposure, or that it completely recovers by the time of the examination (9th day after landing). The latter interpretation is supported by a recent study by Yuan et al. (2018a), who found altered activation in the cerebellum, hippocampus, and visual areas elicited by an active foot tapping fMRI paradigm in a group of volunteers during 70-day HDBR, an effect which was not found a week after HDBR. Also, microgravity-induced changes within the human sensorimotor system are likely to be paradigm-dependent, as was illustrated by our earlier case study (Demertzi et al., 2016) that reported enhanced activation in the SMA during an imaginary tennis task, but not a task involving imagery navigation through a house, after spaceflight.

### Intrinsic Connectivity Contrast Data

Our data-driven approach revealed altered ICC for the plantar stimulation over baseline within the right posterior SMG in cosmonauts after spaceflight in comparison to the control group. This part of the SMG belongs to the temporoparietal junction (TPJ) region which also contains a part of the angular gyrus and the most caudal portion of the superior temporal gyrus. The TPJ region in general is believed to play an important role in the processes of motor adaptation, multisensory integration (Grefkes and Fink, 2005) and bodily self-consciousness (Pfeiffer et al., 2014). Therefore our present finding resonates with the recent results by Van Ombergen et al. (2017d), who studied healthy participants exposed to acute alterations of gravity during a parabolic flight and found a decreased resting-state ICC in the right TPJ, specifically within the angular gyrus. Additionally, their results revealed increased connectivity of this region with the SMG bilaterally. The pSMG area is known to be involved in the processing of vestibular input (Bense et al., 2001), in the perception of being upright (Kheradmand et al., 2013), and in the visual perception of object motion according to the laws of gravity (Indovina et al., 2005); the angular gyrus is associated with coordination of sensory weighting and sensory realignment mechanisms during sensorimotor adaptation (Block et al., 2013). Interestingly, the two regions showing signs of structural connectivity disruption after the spaceflight that were found in a recent diffusion MRI study by Lee et al. (2019) also lie in the white matter below the right TPJ region next to the rpSMG. The involved white matter regions belong to the superior longitudinal fasciculus, the inferior longitudinal fasciculus, and the inferior fronto-occipital fasciculus, which are all important for connecting the parietal and frontal cortices and play a role in sensory integration (Lee et al., 2019).

While interpretations involving the functions listed above are appealing, they should be considered with caution because of the heterogeneity and polyfunctional nature of the TPJ (Lee and McCarthy, 2016; Schuwerk et al., 2017) and because of the lack of exact overlap between the coordinates reported in the discussed studies and the locus of our finding (except for the overlap with results by Indovina et al., 2005). The latter is especially important given the considerable spatial extension of both the TPJ and the SMG. Remarkably, the region in the right inferior parietal cortex (BA40) that in the present study was identified as a region showing not increasing but decreasing connectivity after the spaceflight with the ROI-to-ROI approach, is also located within the rSMG, although at a distance from the cluster revealed by the ICC approach.

### ROI-to-ROI Analysis

#### Decreased Connectivity of the Vestibular Nuclei

Altered connectivity of the vestibular nuclei was among the most predictable results of the current study because of the severe impact of microgravity on the vestibular system, starting with the deconditioned gravity sensing otolith system (Moore et al., 2003; Kornilova et al., 2012; Hallgren et al., 2016). Although the nature of the NBS results does not allow for discussion of any individual connection but only of the entire cluster of the assessed connections of the vestibular nuclei, our data make evident the decreased connectivity of the vestibular nuclei with multiple regions including other parts of the vestibular brain system (operculum, precuneus, inferior parietal cortex) as well as with motor, somatosensory, cerebellar, and visual regions in cosmonauts after spaceflight. Such disintegration cannot be accounted for by the decreased activity in the vestibular nuclei, because it was shown previously that monkeys traveling to space with chronically implanted electrodes exhibited not decreased but increased activity of the vestibular nuclei during gaze test performance both inflight and post-flight (Sirota et al., 1988; Badakva et al., 2000; Slenzka, 2003). Therefore, the more plausible explanation of the relative disconnection of the vestibular nuclei involves down-weighting of the vestibular input in order to reduce the conflict between the information from different sensory modalities (proprioception, visual, vestibular) (Block and Bastian, 2011). This idea is also supported by the co-activation pattern evoked by vestibular stimulation in a recent HDBR study by Yuan et al. (2018b) and by the results of the PF study (Van Ombergen et al., 2017d).

#### Increased Interinsular Connectivity

Interestingly, although the insula is considered to be an important part of the human vestibular cortex, and we found that spaceflight significantly alters connectivity of both insulae and the vestibular nuclei, no modifications of the functional connectivity between these two structures were found (even at a liberal, uncorrected threshold), suggesting that the down-weighting of the vestibular input occurs early within the stream of vestibular information processing. At the same time, the right posterior insula showed increasing connectivity with the left posterior insula and a broader area within the left insular and opercular cortex. Also the right posterior and the left anterior insulae significantly changed their connectivity within the set of regions including the proprioceptive cortex and cerebellum, as revealed by the ROI-level NBS analysis. Given that the insula is believed to play an important role not only in vestibular signal processing but also in proprioception, interoception, pain, sensory integration, motor control, and higher processes such as salience detection, emotion, and speech (Kelly et al., 2012), the increased interinsular connectivity may be hypothesized to be a compensatory aid for motor control in conditions of functional loss of vestibular modality and altered proprioception.

Changes in insular connectivity due to microgravity exposure or its ground-based analogs have been reported previously (Zhou et al., 2014; Demertzi et al., 2016). Both studies revealed a reduced resting-state functional connectivity in the insula: Demertzi et al. (2016) found this effect in the right insula of a single cosmonaut post-flight with the ICC measure, while Zhou et al. (2014) revealed it using the degree of centrality (DC) measure in the left anterior insula of a group of HDBR participants. Both findings seem to be inconsistent with our results exhibiting an intensification of insulae bilateral coupling. Since this difference might rise from the applied measure of connectivity, while the ROI-to-ROI approach is both more sensitive and more focused (and therefore it might neglect the insula’s connectivity with areas not considered in the study), we extracted the ICC values for every participant and condition from the right insula ROI constructed as a 10-mm sphere around the coordinates reported by the 2016 study (*x* = 48, *y* = −6, *z* = 4). These data were further used with the same ANCOVA model as the main dataset, with the same contrast (cosmonauts > controls, post-flight > preflight, plantar stimulation > rest). While there was no significant interaction effect for the ICC connectivity measure, in the majority of the cosmonauts (8 out of 11) the individual ICC changes were positive, including the cosmonaut who was the subject of the earlier case study (Demertzi et al., 2016). Therefore, we may conclude that the discrepancy in the observed direction of insular connectivity alterations most likely reflects the specific features of the task-based vs. baseline resting-state connectivity.

#### Decreased Connectivity of the Cerebellum

Last but not least, the finding of degraded connectivity of the cerebellar regions with multiple areas that belong to the motor, somatosensory, parietal proprioceptive, and visual cortices after long-term spaceflight (revealed by the ROI NBS and individual connection ROI-to-ROI analysis) is highly consistent with the existing literature that identifies the cerebellum as a principal brain structure for human adaptation to gravity (Razumeev and Grigoryan, 1976; Sajdel-Sulkowska, 2013), sensorimotor adaptation in general (Martin et al., 1996a, b; Werner et al., 2010; Galea et al., 2011), motor learning and fine motor control (Della-Maggiore et al., 2009; Tomassini et al., 2011; Manto et al., 2012).

Presumably, the cerebellum should play an important role in the adaptation to microgravity alterations, and regional cerebellar volumes and cerebellar-cortical connectivity are considered as possible predictors of adaptation success in space travelers (Seidler et al., 2015). In line with this idea, a recent voxel-based morphometry (VBM) study in humans revealed massive volumetric gray matter reduction in the cerebellum after a HDBR analog of microgravity that does not imply active motor control adaptation, although not after an actual long-term spaceflight (Koppelmans et al., 2016). No massive gray matter volume changes after the long-term spaceflight were reported by Van Ombergen et al. (2018) as well. In rats, a microgravity-induced ultrastructural plasticity of the cerebellum was found even after a 24-h-long spaceflight (Holstein et al., 1999).

The spaceflight-associated decrement in structural connectivity found by Lee et al. (2019) in the inferior cerebellar peduncles is consistent with the evidence for the functional disconnection of both the cerebellum and the vestibular nuclei we observe in the present study, given that the inferior cerebellar peduncle embraces the link between the vestibular nuclei and the cerebellum (Splittgerber, 2019). However, considering the indirect correspondence between structural and functional connectivity, this analogy should be made with caution.

As for the alterations of functioning, a case study (Demertzi et al., 2016) revealed a reduced resting state connectivity between the left cerebellum and the right motor cortex. Similarly, our task-based data on the altered network of the lobules VI–IX (anterior part of the cerebellar network, ROI-level NBS analysis) included decreased connectivity with both right and left lateral motor cortex regions, although these changes did not survive the connection-wise correction for multiple comparisons. Meanwhile, our data do not corroborate the findings by Cassady et al. (2016), who found increased connectivity between the right OP2 and the cerebellum during HDBR and decreased intercerebellar connectivity after bed rest. Overall, the decreased cortico-cerebellum connectivity within the motor control circuit during the gait-like stimulation found in the present study may imply a higher impact of the controlled vs. automated processes in locomotion, as a result of the microgravity exposure or in the process of readaptation to Earth’s gravity. As suggested by the degrading connection between the anterior cerebellum and the right parietal proprioceptive cortex (BA40 ROI), it also may imply the so-called cerebellum-dependent adaptation, or recalibration of the relationship between sensory input and motor output (Block and Bastian, 2012).

### Connectivity of the Primary Motor and Somatosensory Cortex

Many researchers have found structural plasticity in the primary motor and somatosensory cortex after spaceflight and its analogs. One of the important findings in astronauts reported by Koppelmans et al. (2016) is a focal gray matter increase in the medial paracentral lobule, a region including the representation of the lower limbs within the primary SMC. Li et al. (2015) and Koppelmans et al. (2017b) have also found a volumetric increase of gray matter in the paracentral lobule after HDBR. Animal research has provided a detailed description of the ultrastractural plasticity in the primary motor and somatosensory cortices in rats during and after spaceflight (Belichenko and Krasnov, 1991; DeFelipe et al., 2002; Dyachkova, 2007).

Surprisingly, our data did not reveal any significant alterations in the connectivity of primary motor and somatosensory cortex ROIs. One possible reason for this may be that the gray matter increase reported by Koppelmans et al. (2016) is driven mostly by fluid shift mechanisms, i.e., by the complementary decrease in CSF volume in this region (Van Ombergen et al., 2018) rather than neuroplasticity.

**FIGURE 6:**
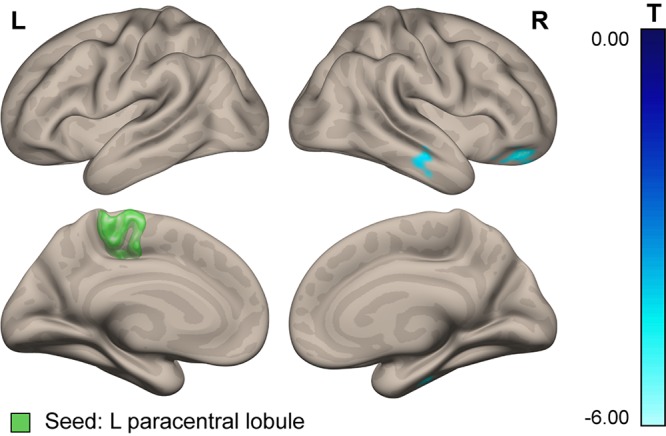
Connectivity of the paracentral lobule. Areas demonstrating altered functional connectivity with the left paracentral lobule evoked by plantar stimulation over the resting baseline in cosmonauts compared to the control group, at post- vs. pre-flight. Blue indicates decreasing connectivity with the seed. Green indicates the location of the seed.

For further clarification, we conducted a seed-to-voxel analysis of the PPI connectivity measures with two seeds in the paracentral lobules (left and right), adopted from T1 MNI ICBM152 and Freesurfer in order to replicate the ROI used by Koppelmans et al. (2016) in their VBM analysis. The results revealed decreased connectivity of the left (but not the right) paracentral lobule with the contralateral frontal pole and middle and inferior temporal gyri in cosmonauts vs. controls at post- vs. pre-flight (see Figure 6 and Table 6). This finding should be considered with caution due to the a posteriori character of the underlying analysis. It also does not rule out the idea that the post-flight alteration of gray matter volume is a mechanical effect. However, it shows that the major counterparts of the connectivity changes that engage the paracentral region lie beyond the set of areas chosen for the ROI-to-ROI analysis in the present study.

**TABLE 6:** Connectivity of the paracentral lobule.

**Seed**	**Resulting cluster center of mass, MNI**			**Cluster size, vx (mm^3^)**	**Cluster *p* uncorr.**	**Cluster *p* FDR-corr.**	FDR *p-*thresh^*^	**PPI value differential effect^+^**	**Region labels**
	***x***	***y***	***z***						
Paracentral lobule, L	24	40	−19	75 (2025)	0.0005	0.006	0.025	−0.13	Frontal pole R Frontal orbital cortex R
Paracentral lobule, L	52	−18	−22	58 (1566)	0.0016	0.011	0.025	−0.12	Inferior temporal gyrus, posterior division R
Middle temporal gyrus, posterior division R									

^+^To aid interpretation, the PPI values (connectivity between the seed and the target region for the task blocks differential from the implicit baseline) were averaged within each cluster. The cosmonaut vs. control, post-flight vs. pre-flight effect on PPI values is presented for illustrative purposes. ^*^Threshold, corrected for the number of seeds.

One possible explanation of this finding is that here we observed a correlate of the previously described phenomenon of an altered lower extremity functional asymmetry in cosmonauts. A change of the leading leg from right before launch to left during the flight was observed in about half of the cosmonauts, according to the data from support reaction registrations in a study of locomotion performance during long-duration spaceflight (Brykov et al., 2015). The plausibility of such an interpretation is increased given the evidence for inversion of other brain functional asymmetries due to support unloading. EEG recordings of presaccadic slow negative potentials (PSNPs) in a dry immersion model have shown that over the course of 7 days of simulated microgravity, the focus of presaccadic negativity shifted to the right hemisphere: the PSNP amplitude sharply decreased in the left and increased in the right hemisphere (Kirenskaya et al., 2006). The authors suggested that because of support unloading and a decrease in proprioceptive input, exposure to microgravity causes a corresponding decrease in the activity of the prefrontal and parietal cortices of the left hemisphere, initially involved in preparation and realization of motor responses. The activation of the right hemisphere in this case could be of compensatory character. The same logic seems to be applicable to our present results.

### Neuroplasticity, Adaptation, and Readaptation

So far, the most unequivocal results advocating for microgravity-induced neuroplasticity in the sensorimotor system of the mammal brain have been obtained with animal models (Belichenko and Krasnov, 1991; DeFelipe et al., 2002; Dyachkova, 2007). As for the human data, even today microgravity-induced neuroplasticity is discussed in the literature only as a highly plausible theoretical speculation not sufficiently supported by empirical evidence (Koppelmans et al., 2017a; Van Ombergen et al., 2017a). The reason for this caution is not only a lack of data, but also the problems of inference. With non-invasive techniques, central neuroplasticity can hardly be disentangled from numerous low-level confounds arising from the upward displacement of the brain within the skull (Roberts et al., 2017), changes in CSF production and reabsorption (Nelson et al., 2014; Kramer et al., 2015), adaptations in cardiovascular functioning (Zhu et al., 2015) and brain hemodynamics (Kawai et al., 2003; Blaber et al., 2013; Taylor et al., 2013).

However, it is very unlikely that behavioral adaptation and motor learning in space travelers is acquired without any modifications in brain structure and function. Modern neuroscience has collected extensive evidence for experience-induced neuroplasticity in adults learning new skills, and such plasticity becomes visible with neuroimaging even in short periods of time (Dayan and Cohen, 2011; Sampaio-Baptista et al., 2018). This research may guide our understanding of spaceflight’s consequences and their reversible nature (Seidler et al., 2015).

Upon returning to Earth, the space crewmembers pass through a readaptation period since the new motor control strategies acquired in microgravity become maladaptive in the terrestrial settings. Transition to another state of the sensorimotor system takes time, and may be traced for up to 2 weeks at least (Mulavara et al., 2010). The time-course of the readaptation demonstrates substantial individual differences and may be affected by a wide range of factors, from genetic to behavioral (Seidler et al., 2015). Therefore, the signs of the neuroplasticity observed in the readaptation period should be considered not only as residual consequences of the long-term microgravity exposure, but as a mixture of effects induced by spaceflight and the subsequent readaptation to Earth. This view is supported by evidence from animal research which shows continuing ultrastructural modifications in the rat somatosensory cortex, especially in terms of the functional activity or degeneration of axonal terminals in the first hours upon reentry to the Earth’s gravity and even 14 days after landing (Dyachkova, 2007). With this consideration, the time period between landing and subsequent MRI examination, for example, the difference of 4 vs. 9 days discussed by Roberts et al. (2017) should be taken into account as an important variable in future research.

The results of the present study perfectly illustrate the inference problem arising from the mixture of adaptation and readaptation processes. Thus, the unchanged fMRI activation pattern elicited by the plantar stimulation may reflect either the preservation of the ‘core’ neural system associated with the reaction to support loading, or the fast and effective recovery of this system in the first days after spaceflight. Similarly, the observed modifications of the connectivity between different sensory inputs utilized by the motor system (vestibular, proprioceptive, visual) may reflect not only impairment in the default motor control connectivity due to the vestibular deprivation and biomechanic factors associated with microgravity, but also a dephasing of the motor control strategies adopted during spaceflight in favor of the neural implementation of ground-based locomotion.

Interestingly, the hypothesis that after a prolonged space flight the human brain features two co-existing neural networks supporting two modes of locomotion — one for 1 g-gravity and one for microgravity — could account for the fact that second-time flyers adapt more quickly and are less prone to microgravity-induced problems (Kozlovskaya et al., 2015). It also encourages a perspective on divergent signs between the task-based and resting-state connectivity alterations highlighted in the previous discussion (the right TPJ results in Van Ombergen et al., 2017d and in the present study; the insula connectivity results in Zhou et al., 2014; Demertzi et al., 2016 and in the present study). The task-based connectivity measures provide information about the current state of the functional brain organ accomplishing some motor activity such as locomotion. At the same time, it is widely believed that resting-state connectivity represents the background activity of large-scale neural networks that become active when triggered by certain stimulations or tasks (Heine et al., 2012). If so, the resting-state connectivity after a space flight provides amalgamated information about all co-existing motor networks, both the one presently used and the one adapted for microgravity conditions and unused in terrestrial settings. Therefore, a comparison of the connectivity data from these two sources may provide an important basis for future insights into the mechanisms of neuroplasticity within the motor system.

### Implications for the Sensory Reweighting Theory

In microgravity, alterations in biomechanics such as modified relationships between the mass of a body part and the force required to move it call for a recalibration of the correspondence between sensory input and motor output (sensory realignment), which is believed to be the function of the cerebellum (Block and Bastian, 2012). At the same time, the otolith afferentation is substantially altered. Not only does it become unreliable, but the otherwise tight coupling of canal-otolith information gets lost, and a conflict in the input from different sensory modalities (vestibular, proprioceptive, and visual) is created (Kornilova and Kozlovskaya, 2003; Davis et al., 2008). The motion sickness syndrome is believed to be a consequence of such sensory mismatch (Reason and Brand, 1975; Schmäl, 2013) and is often experienced by space travelers and described as space motion sickness (Heer and Paloski, 2006; Kornilova et al., 2013).

This sensory conflict calls for a second type of sensorimotor adaptation that involves a recalibration of the relationship between several sensory modalities. Theoretically, this may be dealt with by reweighting the sensory inputs; that is, by downweighting the less reliable modality and prioritizing a more reliable one (Horak et al., 1990). The neural mechanisms of multisensory reweighting remain generally unclear. Reweighting has been studied predominantly in patients with postural pathologies and sensory loss, children and the elderly (e.g., Jeka et al., 2010; Polastri and Barela, 2013), but it has also been suggested as an important mechanism of motor adaptation in astronauts who demonstrate increased reliance on visual and tactile information during and after spaceflight (Reschke et al., 1998; Speers et al., 1998; Clément et al., 2001). Evidence for multisensory reweighting in astronauts has been collected on the basis of kinematic and tactile sensitivity research and subsequent modeling (Speers et al., 1998; Clément et al., 2001; Lowrey et al., 2014).

Given the substantial individual differences between space travelers in their susceptibility to space motion sickness (and therefore in the supposed degree of the proposed sensory reweighting) and the small size of our sample, it may be the case that pronounced neural correlates of sensory reweighting may be found in some but not all cosmonauts. Therefore we examined the role of individual differences and looked for possible correlations between the severity of space motion sickness symptoms and connectivity of the areas changing their connectivity pattern during plantar stimulation in cosmonauts post-flight as shown by the ROI-to-ROI and ICC approaches. The observed correlations suggest the potential importance of connection between the left insula and the right TPJ regions for the neural mechanisms of space motion sickness and sensory reweighting in microgravity. The greater the post- vs. pre-flight difference in connectivity between the two regions, the more pronounced were the symptoms of space motion sickness, which is consistent with the idea of a possible compensatory role of the insula and its connectivity, as discussed above.

### Implications for the Gravitational Motor System Theory

According to the gravitational motor system theory (Kozlovskaya et al., 1988), keeping an upright stance in humans is the function of specialized tonic muscle system mainly comprised of the tonic motor units of the extensors. The support afference from the deep skin mechanoreceptors is considered to be the most important sensory input driving motor control in this system. Empirical evidence comes from both actual spaceflights and ground-based models showing that withdrawal of support entails a reflectory decline of transverse stiffness and a voluntary force of tonic extensor (postural) muscles, limiting their participation in locomotion and increasing the involvement of phasic muscle units (Kozlovskaya et al., 1988, 2007; Shigueva et al., 2015). The prevalence of the flexor over the extensor activity leads to the adoption of a quasi-embryonic flexor posture (Margaria, 1966; Bogdanov and Gurfinkel, 1976) and to a changed pattern of coordinated muscle recruitment (Roy et al., 1996). Support unloading has also been found to result in increased sensitivity of the vestibular system in different types of dry immersion in humans (Kreidich, 2009; Kornilova et al., 2016). Similar increased vestibular excitability has also been revealed in the actual microgravity in primates (Sirota et al., 1988) and in cats and a monkey in parabolic flight (Grigorian et al., 1995). These findings were in line with the hypothesis that the vestibular system normally experiences an inhibitory influence from the support afferent system and activates when the influence is removed (Kreidich, 2009). Such inhibitory modulation may be implemented through inhibitory connections projecting from the cerebellum onto the vestibular nuclei (Ito, 1972).

Our present results are in agreement with this theory. In space travelers we did not find evidence for alterations in brain activation elicited by the support afferentation (gait-like plantar stimulation). This is likely to be a result of the fast recovery of the system processing support afference in cosmonauts within the very first days upon their return to the Earth’s gravity. At the same time, evidence for the down-weighting of the vestibular input was still present on the 9th day upon return when most cosmonauts underwent their post-flight scan. Since in microgravity the support afferent system becomes effectively silent and therefore is not involved in sensory conflicts, we may suppose that it is also minimally involved in sensory re-weighting, unlike the vestibular system which produces massive but unreliable sensory signals during space flight. We may further suppose that the fast recovery of the support afferent system’s activity upon return to Earth is accompanied by the restoration of its inhibitory modulation on the vestibular system, which in turn slows down the recovery of the vestibular function. Testing this hypothesis may become a prospective direction of future behavioral and neuroimaging research.

## Conclusion

Our data show changes in functional brain connectivity specific for a plantar stimulation task in a group of the cosmonauts after long-term spaceflight as compared to a control group. The observed alterations included a disconnection of the vestibular nuclei, the superior part of the right supramarginal gyrus, and the cerebellum from a set of motor, somatosensory, visual, and vestibular areas. Increased connectivity was found between the left and right insulae as well as between the part of the right posterior supramarginal gyrus within the TPJ region and the rest of the brain. A post- to pre-flight difference in connectivity between the latter area in the right posterior temporal cortex and the left anterior insula demonstrated a correlation with the severity of space motion sickness symptoms. At the same time, no alterations were found in activation elicited by the gait-like plantar stimulation. The findings cannot be attributed solely to the lasting effects of long-term microgravity exposure since such effects are contaminated by the readaptation to Earth’s gravity that took place between the landing and the post-flight MRI session. Nevertheless, the results suggest implications for the multisensory reweighting and gravitational motor system theories, generating hypotheses to be tested in future research.

## Ethics Statement

This study was approved as a part of the Brain DTI project by Committee of Biomedicine Ethics of the Institute of Biomedical Problems of the Russian Academy of Sciences and the Human Research Multilateral Review Board (HRMRB) according to the 18th World Medical Assembly of Helsinki, Finland, June 1964, amended by the 41st Assembly, Hong Kong, September 1989. All participants gave written informed consent for the study at enrollment.

## Author Contributions

EP helped with the design the study, made the fMRI data analysis, and wrote the draft of manuscript. InN, AG, IR, and SJ coordinated the study and participated in conducting research. AR and LL conducted fMRI procedure and contributed to analyzing the experiment results. FW, ET, and IK participated in conceiving and designing of the idea. LC contributed to methodology and participated in critically revising the manuscript. IvN and LK contributed in the part of observations and processing of vestibular function state. BJ and AVO helped with the design the study, took part in conducting research. SL and JS participated in discussions and critically revising the manuscript. EM and VS coordinated the study, participated in critically revising the manuscript. All the authors took part in writing, review and editing and read and approved the current manuscript.

## Conflict of Interest Statement

The authors declare that the research was conducted in the absence of any commercial or financial relationships that could be construed as a potential conflict of interest.

## Footnotes

1 www.fil.ion.ucl.ac.uk/spm

2 http://mrtools.mgh.harvard.edu/

3 http://www.nitrc.org/projects/conn

4 http://www.nitrc.org/projects/artifact_detect
