# Supplementary material for: Alterations of Functional Brain Connectivity After Long-Duration Spaceflight as Revealed by fMRI
Source: Front Physiol. 2019 Jul 4;10:761. doi: 10.3389/fphys.2019.00761 (PMC6621543; doi:10.3389/fphys.2019.00761)
Supplement: Supplementary file 1 [file Table_1.DOCX]

Space Motion Sickness Questionnaire (ANKETA)

1. Questions to be answered by the cosmonauts before the space flight:

1.1. Are you susceptible to motion sickness (when using means of transportation, at sea, on amusement park attractions, during acrobatics, etc.)?

1.2. When you use means of transportation, does the landscape swiftly moving outside provoke or increase symptoms of motion sickness?

1.3. How did you feel during vestibular expert tests, vestibular training and Kepler parabolic flights?

1.5. How did you feel during optokinetic exposures during experimental testing?

1.6. Do head movements deteriorate your gait and standing (the Romberg posture)?

2. Questions to be answered by the cosmonauts after the space flight.

Consider whether you experienced any of the following reactions after your space flight:

2.1. Difficulties with spatial orientation (loss of the concept of top-bottom, the feeling of the body tilting or its inversion)?

2.2. Illusions of body movement (linear, rotational or combined); if so, for which body axes did these illusions predominate?

2.3. Dizziness (spontaneous, when tilting the head in the sagittal or frontal planes, when turning the head around the longitudinal axis of the body)?

2.4. Impaired coordination of arm, leg or body movements?

2.5. Difficulties in gaze holding and tracking of visual objects (without any movements, with head movements, with eye movements, during reading, during long-term observation of visual objects in the center and at the periphery of the visual field)?

2.6. Nausea (mild or severe, lasting for how many hours or days) and vomiting (once or repeatedly, on what day after the space flight)?
